# Transplantation of human neural stem cells transduced with Olig2 transcription factor improves locomotor recovery and enhances myelination in the white matter of rat spinal cord following contusive injury

**DOI:** 10.1186/1471-2202-10-117

**Published:** 2009-09-22

**Authors:** Dong H Hwang, Byung G Kim, Eun J Kim, Seung I Lee, In S Joo, Haeyoung Suh-Kim, Seonghyang Sohn, Seung U Kim

**Affiliations:** 1 Brain Disease Research Center, Institute for Medical Sciences, Ajou University School of Medicine, Suwon, Korea; 2 Department of Neurology, Ajou University School of Medicine, Suwon, Korea; 3 Department of Anatomy, Ajou University School of Medicine, Suwon, Korea; 4 Laboratory of Cell Biology, Institute for Medical Sciences, Ajou University School of Medicine, Suwon, Korea; 5 Medical Research Institute, Chungang University School of Medicine, Seoul, Korea; 6 Division of Neurology, Department of Medicine, UBC Hospital, University of British Columbia, Vancouver, Canada

## Abstract

**Background:**

Contusive spinal cord injury is complicated by a delayed loss of oligodendrocytes, resulting in chronic progressive demyelination. Therefore, transplantation strategies to provide oligodendrocyte lineage cells and to enhance the extent of myelination appear to be justified for spinal cord repair. The present study investigated whether transplantation of human neural stem cells (NSCs) genetically modified to express Olig2 transcription factor, an essential regulator of oligodendrocyte development, can improve locomotor recovery and enhance myelination in a rat contusive spinal cord injury model.

**Results:**

HB1.F3 (F3) immortalized human NSC line was transduced with a retroviral vector encoding *Olig2*, an essential regulator of oligodendrocyte development. Overexpression of Olig2 in human NSCs (F3.Olig2) induced activation of NKX2.2 and directed differentiation of NSCs into oligodendrocyte lineage cells *in vitro*. Introduction of Olig2 conferred higher proliferative activity, and a much larger number of F3.Olig2 NSCs were detected by 7 weeks after transplantation into contused spinal cord than that of parental F3 NSCs. F3.Olig2 NSCs exhibited frequent migration towards the white matter, whereas F3 NSCs were mostly confined to the gray matter or around the lesion cavities. Most of F3.Olig2 NSCs occupying the spared white matter differentiated into mature oligodendrocytes. Transplantation of F3.Olig2 NSCs increased the volume of spared white matter and reduced the cavity volume. Moreover, F3.Olig2 grafts significantly increased the thickness of myelin sheath around the axons in the spared white matter. Finally, animals with F3.Olig2 grafts showed an improvement in the quality of hindlimbs locomotion.

**Conclusion:**

Transplantation of NSCs genetically modified to differentiate into an oligodendrocytic lineage may be an effective strategy to improve functional outcomes following spinal cord trauma. The present study suggests that molecular factors governing cell fate decisions can be manipulated to enhance reparative potential of the cell-based therapy.

## Background

Traumatic spinal cord injury (SCI) results in severe and permanent neurological deficits. However, there is no single effective therapeutic option to improve functional outcomes. Intense research efforts, employing a rodent model of contusive injury which closely mimics human SCI, have identified that the pathology in the white matter incurred by injury is closely associated with the degree of functional deficits [1-3]. One of the important pathological processes in the white matter is a chronic and progressive demyelination of the spared axons [4-7], which occurs primarily due to delayed and widespread apoptosis of the oligodendrocytes [8,9]. Absence of myelin sheath and resultant exposure of potassium channels lead to a failure of electrical conduction through spared axons, contributing to chronic functional deficits following SCI [10]

From these observations, transplantation strategies to provide cells capable of myelinating axons and enhance remyelination seem to be well justified for spinal cord repair. Transplantation of oligodendrocyte progenitor cells or glial progenitor cells derived from embryonic or neural stem cells promoted functional recovery in SCI animal models [11-13]. Furthermore, a large part of behavioral gains following grafts of murine neural stem cells without lineage restriction has recently been attributed to an enhanced myelination in the spared white matter [14,15]. These studies indicate that enhancing myelination by transplantation of stem/progenitor cells is a promising approach to improve functional outcomes for patients suffering from SCI.

An increasing number of molecular factors that govern the fate determination of neural cells during development have been identified [16-18]. Manipulation of appropriate factors may facilitate differentiation of transplanted cells to a desired lineage. The basic HLH transcription factor Olig is a key regulator for the differentiation of oligodendrocyte lineage cells during development [19-21]. Olig2, one of the Olig family, is more highly expressed in the ventral spinal cord during early developmental period of human fetus and may play a crucial role in the differentiation of oligodendrocytes in the spinal cord [22]. In the present study, we overexpressed *Olig2 *gene in stable immortalized human neural stem cells (NSCs), which have been widely employed to repair the CNS in various experimental models of neurological disorders [23-28]. Here we show that overexpression of Olig2 transcription factor directed differentiation of human NSCs exclusively into an oligodendrocyte lineage *in vitro*. We also report that transplantation of Olig2 overexpressing human NSCs improved locomotor function and increased the extent of myelination of spared white matter in a rat SCI model.

## Results

### Characterization of NSCs transduced with Olig2 transcription factor

**Figure 1:**
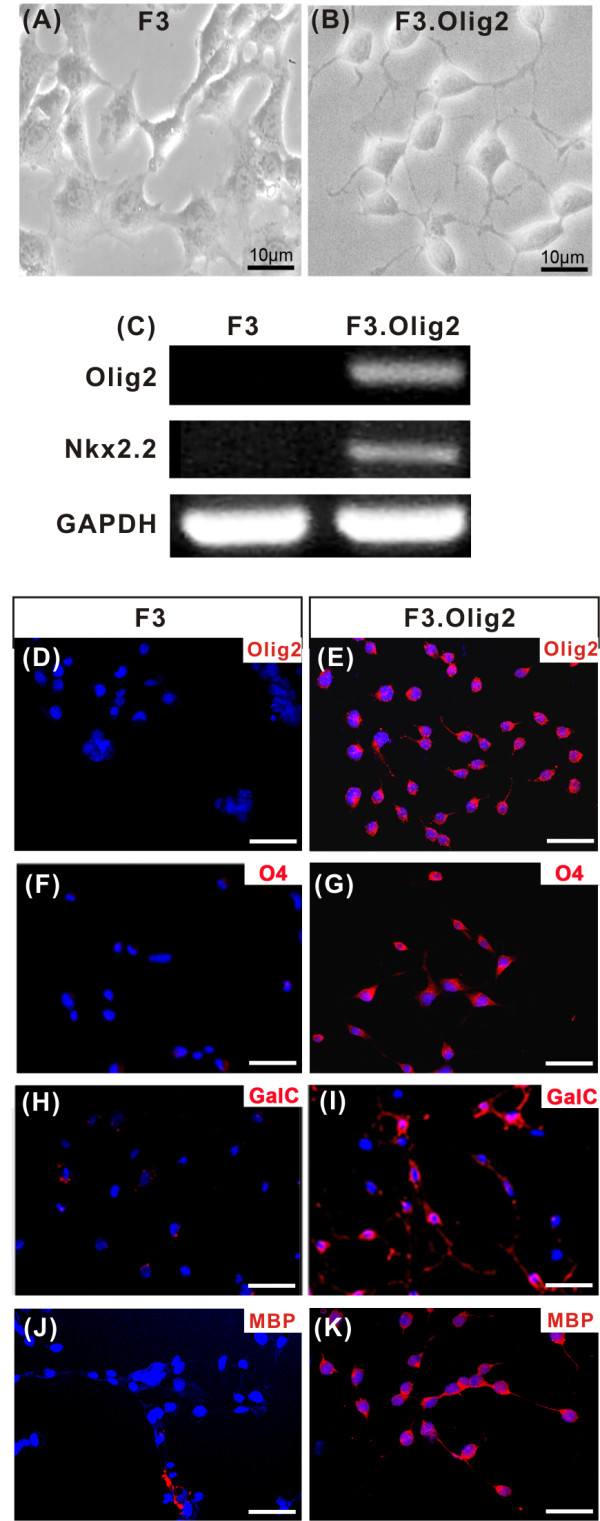
**Characterization of human neural stem cells (NSCs) transduced with Olig2 transcription factor**. (A, B) Phase contrast images of the parental NSCs (F3) and F3.Olig2 NSCs. (C) Comparison of gene expression by RT-PCR analysis between F3 and F3.Olig2 NSCs. (D, E) Confirmation of Olig2 transcription factor expression in F3.Olig2 cells (E). Olig2 expresssion was not observed in F3 cells (D). (F-K) *In vitro *immunocytochemical detection of oligodendrocyte lineage markers as indicated. Cells were grown on coverslips in DMEM with 2% FBS for 5 days and then fixed with 4% paraformaldehyde. (F, H, J) F3 cells, (G, I, K) F3.Olig2 cells. GalC = galactocerebrosidase, MBP = myelin basic protein. Scale bar = 50 μm.

Human NSC line overexpressing Olig2 (F3.Olig2) was generated by retroviral transduction of parental F3 human NSC line with the full length coding region of bHLH transcription factor Olig2. Introduction of Olig2 gene resulted in a change of cellular morphology (Figure 1A, B). F3.Olig2 cells exhibited multiple thin-branched cytoplasmic processes in a phase-contrast image, whereas F3 NSCs showed polygonal shape without the branched processes. RT-PCR analysis confirmed expression of Olig2 mRNA in F3.Olig2 cells. Nkx2.2, a transcription factor which directly regulates the differentiation and maturation of oligodendrocytes [29], was not expressed in F3 parental NSCs but newly expressed after introduction Olig2 gene (Figure 1C). Olig2 expression at the protein level was also confirmed by immunocytochemistry (Figure 1D, E).

Immunocytochemical analysis of phenotypic expression showed that essentially all of F3.Olig2 cells expressed oligodendrocytic lineage markers such as O4 and GalC (Figure 1G, I), whereas the expression of those markers was rarely observed in F3 NSCs (Figure 1F, H). F3.Olig2 cells also expressed myelin basic protein (MBP) (Figure 1J, K), indicating that F3.Olig2 cells could differentiate into myelinating mature oligodendrocytes. These results indicate that ectopic overexpression of Olig2 transcription factor in NSCs turns on transcriptional machinery for the oligodendrocytic cell fate and forces F3 human NSCs to differentiate into oligodendrocytic lineage path. However, F3.Olig2 cells did not show differentiation into mature astrocytes or neurons (See Additional file 1).

### Transplantation of NSCs transduced with Olig2 transcription factor into contused rat spinal cord

**Figure 2:**
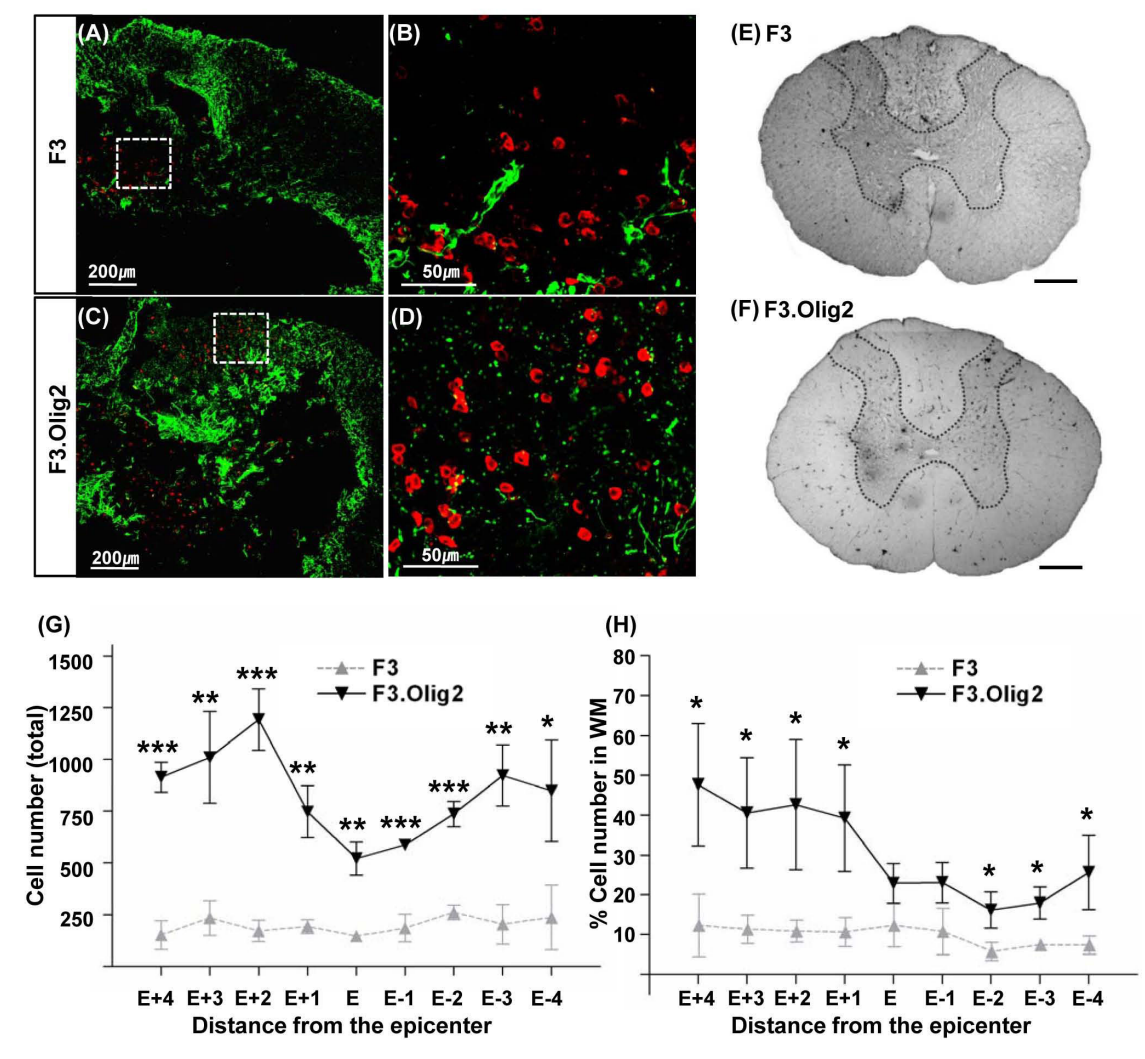
**Transplantation of Olig2 overexpressing human NSCs after contusive spinal cord injury**. (A-D) Immunofluorescence staining of the transverse sections close to the epicenter at 2 weeks after transplantation of F3 (A, B) and F3.Olig2 (C, D) cells. Grafted human NSCs were identified by immunoreactivity against human specific mitochondria (red). GFAP staining (green) was performed to depict the appearance of spinal cord lesions. Photomicrographs in B and D are magnified images of the boxed regions in A and C, respectively. (E, F) Bright field microscopic images of the transverse sections located at 2 mm rostral to the epicenter at 7 weeks after transplantation of F3 (E) and F3.Olig2 (F) NSCs. Many F3.Olig2 cells were observed in the white matter at this time point. Dotted lines indicate boundaries of the gray matter. Scale bar = 500 μm. (G) The number of human mitochondria positive cells was stereologically counted in the spinal cord sections from the epicenter to 4 mm rostral and caudal regions at 1 mm interval. (H) The percentage grafted cells in the ventrolateral white matter versus total number of grafted cells in the entire transverse sections. * = *p *< 0.05, ** = *p *< 0.01, and *** = *p *< 0.001 by unpaired T test at each distance from the epicenter. E represents epicenter. Error bars indicate mean ± SEM. N = 4 animals per group.

Animals received vehicle injection or cellular grafts 7 days after contusive SCI. At 2 weeks after transplantation, grafted cells that were immunoreactive to human specific mitochondria (either F3 or F3.Olig2) were found in clusters mostly around the lesion sites (Figure 2A-D). At 7 weeks, both F3 and F3.Olig2 cells were still observed in the contused spinal cord (Figure 2E, F). Unexpectedly, the number of F3.Olig2 cells was markedly larger than that of F3 NSCs along the rostrocaudal extent of the lesioned spinal cord at this time point. The number of F3.Olig2 cells was highest around the rostral injection site (2 mm rostral to the epicenter) (Figure 2G). Differences in the number of surviving grafted cells between F3 and F3.Olig2 cells were statistically significant over the different rostrocaudal regions from the epicenter (*p *< 0.001 by repeated measures two-way ANOVA). At this time point, distribution of grafted F3.Olig2 cells was different from that of F3 cells. Many of grafted F3.Olig2 cells seemed to migrate from the injection site and occupied the white matter. In contrast, most F3 cells without Olig2 overexpression were still positioned inside the gray matter or around the lesion cavities, with only about 10% of them observed in the ventrolateral white matter (Figure 2H). Differences in the pattern of distribution were most apparent at the rostral regions, where the proportions of grafted cells within the white matter were 5 fold higher in F3.Olig2 NSCs (Figure 2F). The percent grafted cells within the white matter versus total human mitochondria positive cells in the entire cross section was significantly higher in F3.Olig2 than F3 NSCs (*p *< 0.001, by repeated measures two-way ANOVA). Taken together, these results suggest that overexpression of Olig2 transcription factor leads to an increase in the number of surviving grafted cells in the contused spinal cord. In addition, they also indicate that grafted cells with Olig2 transcription factor possess propensity for migration towards the white matter.

**Figure 3:**
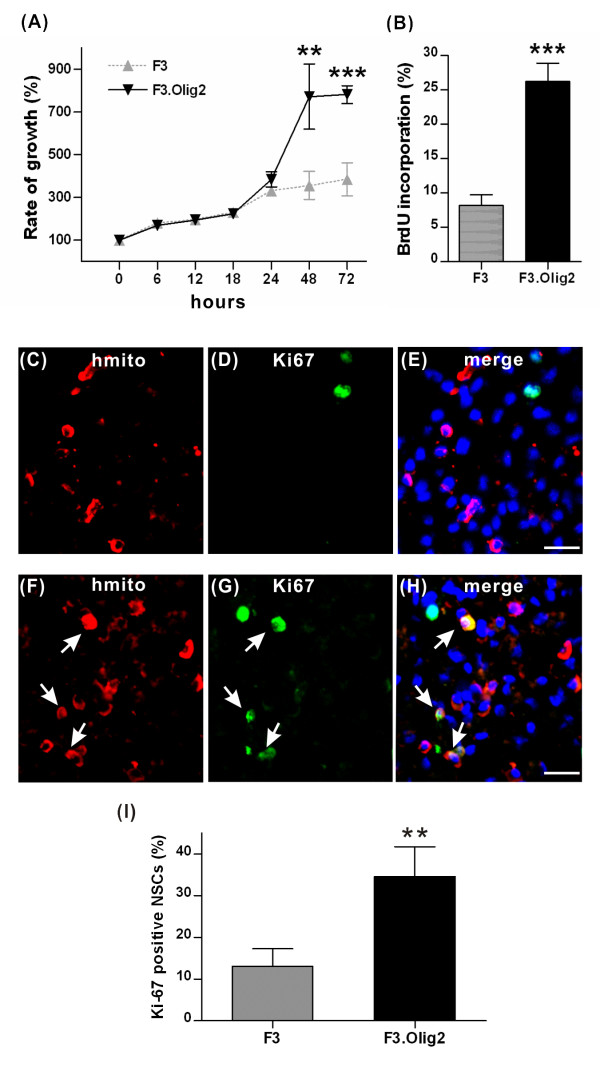
**Comparison of proliferative capacity between F3 and F3.Olig2 human NSCs**. (A) Cell proliferation assay measured by Cell Counting Kit-8. Cells were grown in a 96-well plate and the viability was measured at different time points after initial culture. ** = *p *< 0.01, *** = *p *< 0.001 by unpaired T test. Error bars indicate mean ± SD. N = 4 replicate experiments. (B) BrdU incorporation assay. Cells were grown on a 9 mm coverslip for 36 hours and BrdU was added for 2 hours. *** = *p *< 0.001 by unpaired T test. Error bars indicate mean ± SD. N = 3 coverslips for each condition. (C-H) Representative images of transverse spinal cord sections doubly stained with human mitochondria (red) and Ki67 (green) at 2 weeks after transplantation. A majority of grafted F3 cells (C-E) did not express Ki67, whereas the nuclei of F3.Olig2 grafted cells (F-H) were frequently colocalized with Ki67 (arrows). The nuclei were visualized by DAPI (blue) (E, H). Scale bar = 20 μm. (I) Quantification of the percent grafted cells containing Ki67 positive nuclei. Error bars indicate mean ± SD. ** = *p *< 0.01 by unpaired T test. N = 3 and 4 animals for F3 and F3.Olig2 groups, respectively.

A recent study indicated that Olig2 transcription factor plays a critical role in the regulation of NSC proliferation [30]. It is conceivable, therefore, that the presence of higher number of F3.Olig2 cells may be due to a heightened proliferative activity induced by ectopic expression of Olig2 transcription factor. Consistent with this idea, growth of F3.Olig2 cells *in vitro *was significantly accelerated after 24 hours in culture (Fig 3A). Differences in cell proliferation were highly significant at both 48 and 72 hours (*p *< 0.001 by unpaired T test). BrdU incorporation index was also obtained as a marker of cellular proliferation at 36 hours when the slope of proliferation curve was steepest (Fig 3B). F3.Olig2 cells incorporated proliferation marker BrdU far more frequently than F3 cells (*p *< 0.001). More importantly, grafted F3.Olig2 NSCs at 2 weeks after transplantation were positive for proliferation marker Ki67 more frequently (> 2.5 fold) than F3 (Figure 3C-I) (*p *< 0.01). These results indicate that Olig2 overexpression can increase the size of proliferative progenitor pool and the higher proliferative potential may explain the larger number of F3.Olig2 cells in the injured spinal cord at 7 weeks after transplantation.

### Differentiation of F3.Olig2 cells in the contused spinal cord

**Figure 4:**
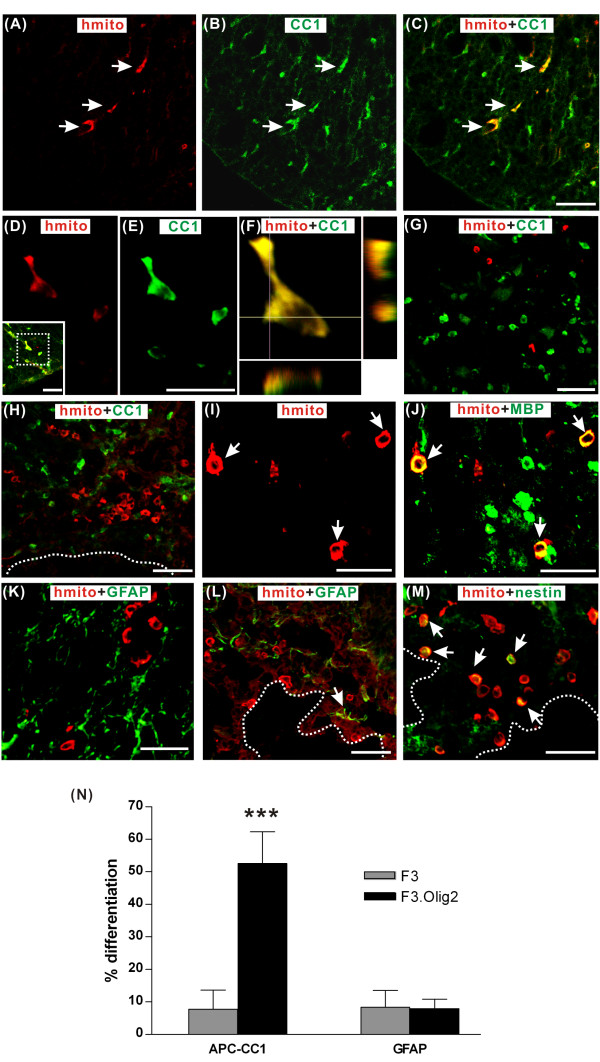
**Phenotypic differentiation of human NSCs transduced with Olig2 transcription factor at 7 weeks following transplantation into contused spinal cord**. Grafted cells were detected by immunoreactivity against human mitochondrial antigen (red). (A-C) F3.Olig2 human NSCs positioned in the spared white matter (A) were colocalized with APC-CC1 (CC-1) (B). Merged cells were shown in yellow (C). Arrows indicate the same cells in the three images. (D-E) Magnified images of a grafted F3.Olig2-derived CC1 positive cell. (Inset) a low magnification merged image showing the location of the magnified cell close to the outer rim of the spared white matter. (F) A 3D reconstruction image of the double positive cells in (D, E). (G) F3 NSCs in the white matter. In contrast to F3.Olig2 cells, F3 cells were very infrequently observed in the white matter and very few of them were positive for CC1. (H) In contrast to F3.Olig2 NSCs in the spared white matter, most of F3.Olig2 cells that remained in the gray were not positive for CC1. (I-J) F3.Olig2 NSCs in the white matter expressed myelin basic protein (MBP). Arrows indicate the same cells. (K) Most of the F3.Olig2 cells in the white matter did not differentiated into astrocytes. (L) Only a small proportion of F3.Olig2 or F3 NSCs in the gray matter were positive for GFAP (arrow) (M) Many F3.Olig2 or F3 cells around the lesion cavity still expressed NSC marker nestin (arrows). Dotted lines in (L-M) indicate the margins of lesion cavities. All scale bars = 20 μm. (N) Quantification of percent NSCs that differentiated into oligodendrocytes (CC1) or astrocytes (GFAP). Error bars indicate mean ± SD. *** = *p *< 0.001 by unpaired T test. N = 4 animals for each group.

Differentiation of grafted F3 or F3.Olig2 NSCs was examined at 7 weeks after transplantation. F3.Olig2 cells identified by human specific mitochondria that migrated to the spared white matter were colocalized with oligodendrocyte marker APC-CC1 (Figure 4A-F). In contrast, as shown in Figure 2C, F3 NSCs without Olig2 transcription factor were rarely found in the spared white matter, and even a majority of F3 cells that migrated to the white matter did not express APC-CC1 (Figure 4G). Interestingly, F3.Olig2 cells that remained in the gray matter or around the lesion cavity showed different behaviors (Figure 4H). They were usually found in aggregation and differentiation into APC-CC1 positive oligodendrocytes was very infrequently observed when compared to those cells positioned in the white matter. Quantification showed that 52.6 ± 9.8% of grafted F3.Olig2 NSCs were colocalized with APC-CC1 as compared to 7.7 ± 5.9% for parental F3 cells (Figure 4N) (*p *< 0.001). When the measurement was confined to the white matter, 84.9 ± 2.2% of F3.Olig2 NSCs were doubly positive for human specific mitochondria and APC-CC1. F3.Olig2 NSCs in the spared white matter also express MBP (Figure 4I-J), a cell type specific marker for myelinating oligodendrocytes, suggesting that F3.Olig2 cells in the spared white matter differentiated into mature oligodendrocytes that were capable of producing myelin. Consistent with these results, most F3.Olig2 NSCs in the white matter did not colocalize with astroglial marker GFAP (Figure 4K). In the gray matter, a small proportion of F3.Olig2 NSCs expressed GFAP. F3 NSCs in the gray matter showed a very similar extent of differentiation into astrocytes (Figure 4L). Overall, approximately 10% of both F3 and F3.Olig2 cells differentiated into astrocytes (Figure 4N). Neuronal differentiation was rarely observed in either F3 or F3.Olig2 NSCs in the gray matter (data not shown). These findings suggested that the majority of F3.Olig2 or F3 NSCs in the gray matter remained undifferentiated. Indeed, many of F3 or F3.Olig2 NSCs in the gray matter, especially around the lesion cavities, were still positive for immature NSC marker nestin (Figure 4M).

### Transplantation of F3.Olig2 NSCs increased the volume of spared white matter and enhanced tissue sparing

**Figure 5:**
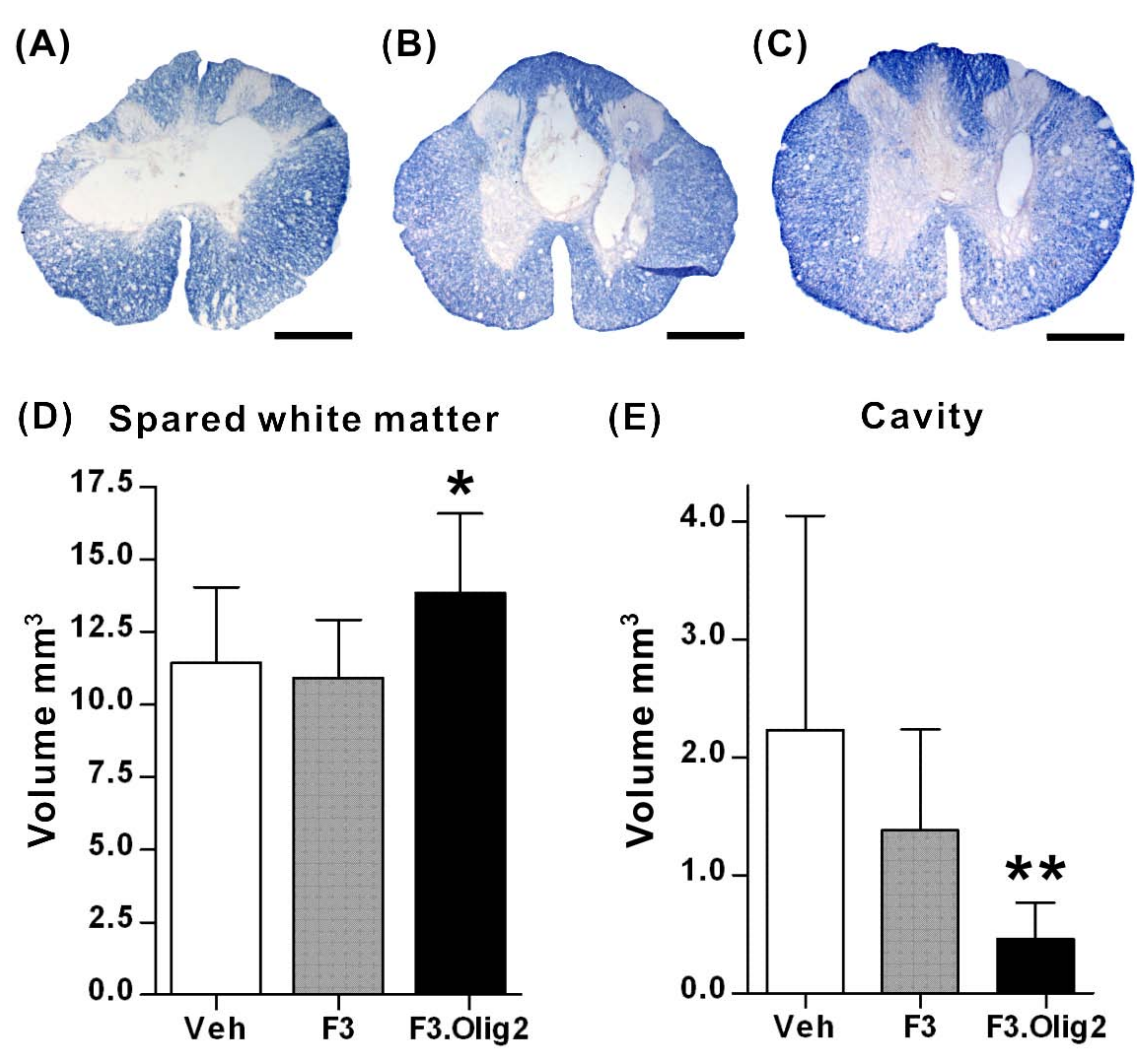
**Volumes of the spared white matter and lesion cavities**. (A-C) Representative images of erichrome-stained spinal cord sections at 1 mm caudal to the epicenter. (A) Vehicle (Veh), (B) F3, (C) F3.Olig2 groups. Scale bar = 500 μm. (D) Comparison of volumes of the spared white matter. (E) Comparison of volumes of lesion cavities. Error bars indicate mean ± SD. N = 11, 12, and 12 for Vehicle, F3, and F3.Olig2 groups, respectively. * = *p *< 0.05, ** = *p *< 0.01 by one-way ANOVA followed by Tukey's *posthoc *analysis.

The areas of myelinated white matter were measured in the eriochrome-stained transverse spinal cord sections (Fig 5A-C), and the volume of spared white matter was calculated using Cavalieri's Principle. Transplantation of F3 NSCs did not affect the mean volume of spared white matter compared to Vehicle group (11.4 ± 2.6 mm^3 ^in Vehicle and 10.9 ± 2.0 mm^3 ^in F3 groups). In contrast, F3.Olig2 grafts significantly increased the volume of spared white matter compared to Vehicle and F3 groups (13.8 ± 2.7 mm^3 ^in F3.Olig2 group; *p *< 0.05 by one-way ANOVA followed by Tukey's *posthoc *analysis) (Figure 5D). We also measured the volume of cystic cavities, and found that transplantation of F3.Olig2 NSCs significantly reduced the volume of cystic cavities (2.2 ± 1.8 mm^3 ^in Vehicle, 1.4 ± 0.9 mm^3 ^in F3, and 0.5 ± 0.3 mm^3 ^in F3.Olig2 group) (Figure 5E). One-way ANOVA revealed significant differences in the volume of cavities between the three groups (*p *< 0.01), and Tukey's *posthoc *analysis showed a significant difference only between Vehicle and F3.Olig2 groups (*p *< 0.01).

### Extent of myelination in the spared white matter

**Figure 6:**
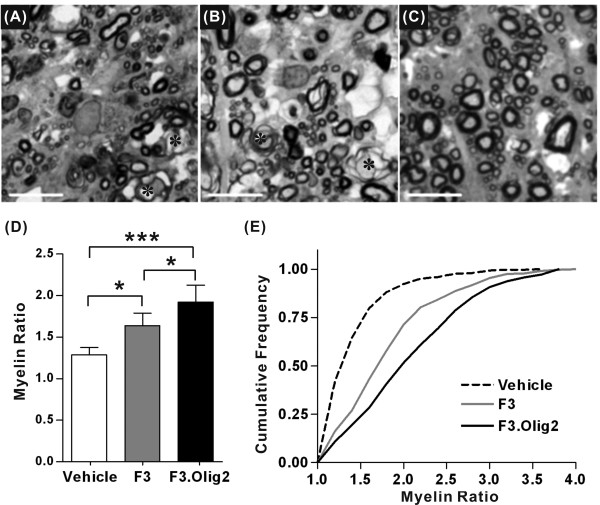
**Measurement of myelin thickness in the spared white matter**. (A-C) Representative images of the ventral white matter in toluidine-blue stained semithin sections from (A) Vehicle, (B) F3, and (C) F3.Olig2 groups. Asterisks indicate degenerating axons. Scale bar = 10 μm. (D) Comparison of the mean myelin ratio. N = 3, 6, and 7 animals for Vehicle, F3, and F3.Olig2 groups, respectively. Error bars represent mean ± SD. * = *p *< 0.05, *** = *p *< 0.001, by one-way ANOVA followed by Tukey's *posthoc *analysis. (E) Cumulative frequency histogram of the myelin ratio. The number of axons analyzed was 692, 1311, and 1348 in Vehicle, F3, and F3.Olig2 groups, respectively.

The observation that F3.Olig2 NSCs in the white matter differentiated into mature oligodendrocytes and that transplantation of F3.Olig2 NSCs increased the volume of spared white matter led us to examine the extent of myelination in the white matter. Thickness of individual myelin sheaths and diameter of the axons were measured in semithin (1 μm) spinal cord sections stained with toluidine blue (3 rats and 692 axons in Vehicle, 6 and 1311 in F3, 7 and 1348 in F3.Olig2 groups, respectively). In toluidine blue stained transverse sections, regions devoid of myelinated axons were frequently observed in the white matter after SCI (Figure 6A). Such demyelinated regions were less frequent in animals with F3.Olig2 grafts (Figure 6A-C). Degenerating axons were also less frequently observed in F3.Olig2 group. We found that the mean myelin ratio was higher in animals with F3 cells compared to Vehicle group (Figure 6E). Transplantation of F3.Olig2 cells further increased the mean myelin ratio. One-way ANOVA revealed a significant group effect on the thickness of myelin sheath (*p *< 0.001). Tukey's *posthoc *analysis showed significant differences between F3.Olig2 and Vehicle or F3 groups (*p *< 0.001 or *p *< 0.05, respectively), and the difference between F3 and Vehicle groups was also significant (*p *< 0.05). Distribution of the myelin ratio in F3.Olig2 group was most shifted to the right compared to the other groups in the cumulative frequency histogram (Figure 6E), indicating that a higher proportion of axons were ensheathed by thicker myelin in animals with F3.Olig2 grafts.

### Transplantation of F3.Olig2 NSCs improves locomotor recovery

**Figure 7:**
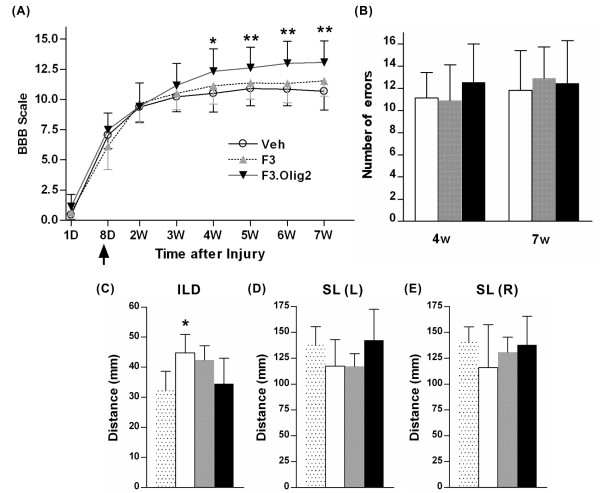
**Transplantation of F3.Olig2 NSCs improves locomotor recovery**. (A) Comparison of BBB locomotor scale. An arrow indicates the time point when transplantation was performed. N = 11, 12, and 12 for Vehicle (Veh), F3, and F3.Olig2 groups, respectively. Error bars represent mean ± SD. * = *p *< 0.05, ** = *p *< 0.01 by repeated measures two-way ANOVA followed by *posthoc *Tukey's analysis. (B) Grid walk. The number of hindpaw placement errors on grid per run was counted at 4 and 7 weeks (4w and 7w, respectively) after transplantation. N = 9, 8, and 9 for Vehicle, F3, and F3.Olig2 groups, respectively. Error bars represent mean ± SD. (C-E) Foot print analysis. (C) Interlimb distance (ILD) or base of support. (D) Stride length (SL) of the left hindlimb. (E) SL of the right hindlimb. N = 5, 4, 5, and 5 for sham, Vehicle, F3, F3.Olig2 groups, respectively. Error bars represent mean ± SD. * = *p *< 0.05 compared to sham. For B - E, stippled, white, gray, and black bars represent sham, Vehicle, F3, and F3.Olig2 groups, respectively.

BBB locomotor rating scale was used to assess the extent of locomotor recovery. Most animals were not able to move their hindlimbs or showed only slight movement of their hip and/or knee joints immediately after contusive injury at the T9 segment. The locomotor deficits were rapidly recovered during the first two weeks regardless of treatment (Figure 7A). The average locomotor scale of the animals with vehicle injection or transplantation of F3 hNSCs began to plateau at 3 weeks and did not show remarkable changes until 7 weeks after injury, the last time point measured. Animals with F3.Olig2 grafts continuously improved locomotor score even after 3 weeks of injury to the extent where they could regain coordination between the fore- and hindlimbs with almost consistent weight-supported plantar steps (average BBB scale 12.8). Repeated measures two-way ANOVA revealed a significant treatment effect over time (*p *< 0.05). Tukey's *posthoc *analysis at each time point showed significant differences in the mean BBB score from 4 weeks after injury (*p *< 0.05 at 4 and 5 weeks; *p *< 0.01 at 6 and 7 weeks). We also measured the number of paw placement errors in grid walk, which is generally regarded as more challenging to injured animals than open field locomotion. Although sham operated animals crossed the grid runway with very few errors, injured animals made more than ten errors per each run (Figure 7B), suggesting that spinal injury severely compromised the sensorimotor integration of the hindlimbs. Transplantation of either F3 or F3.Olig2 cells did not significantly reduce the number errors on grid.

The quality of hindlimbs movement during locomotion was also measured using footprint analysis. Spinal injury resulted in an increase of the distance between the two hindlimbs (interlimb distance) by about 40% (Figure 7C). Transplantation of F3 cells slightly reduced the distance, and the animals with F3.Olig2 grafts almost completely normalized the interlimb distance (*p *< 0.05). The animals with spinal injury also exhibited shorter stride length in both limbs than sham operated rats. Transplantation of F3.Olig2 cells tended to restore normal stride length, although the difference was not statistically significant (Figure 7D, E). These results suggest that grafting hNSCs transduced with Olig2 transcription factor into the contused spinal cord enhances recovery of open field locomotion and improves quality of the hindlimbs movement during locomotion.

## Discussion

In the present study, we demonstrated that transduction of human NSCs with Olig2 transcription factor induces the expression of various phenotypic markers of the oligodendrocyte *in vitro*. The Olig transcription factors (Olig1 and Olig2) are required for the phenotypic determination of oligodendrocytes during development. Genetic ablation of Olig transcription factors resulted in the absence of oligodendrocytes and motor neurons [31-33]. In the spinal cord, Olig2 seems to play a more crucial role for the specification of oligodendrocyte phenotype, since Olig2 expression in the early spinal cord is higher than that of Olig1 [19,20,22]. In the present study, overexpression of Olig2 in the human NSCs led to an activation of NKX2.2, which is known to initiate the expression of oligodendrocyte-specific proteins in collaboration with Olig2 [29,34]. This finding is consistent with the recent reports that overexpression of Olig2 transcription factor is sufficient to induce the expression of Nkx2.2 and differentiation of neural stem/progenitor cells into oligodendrocytes [35,36]. Taken together, these results indicate that forced expression of Olig2 is sufficient to activate the cellular machinery in NSCs that favors phenotypic differentiation into oligodendrocytes.

Although F3.Olig2 cells in the white matter differentiated into APC-CC1 positive oligodendrocytes, the same cells that remained in the gray matter exhibited different biological behaviors. F3.Olig2 cells in the gray matter very infrequently differentiated into mature oligodendrocytes or any other neural cells. F3 NSCs, which are mostly confined around the lesioned areas, also showed very poor differentiation potential. This phenomenon indicates that lesion environment of the spinal cord (especially gray regions around the lesion cavities in this study) inhibits differentiation of transplanted cells as previously reported [37,38]. In F3.Olig2 cells that do not migrate towards the white matter, genetic modification of differentiation properties may not be sufficient to override the influence of lesion environment. Alternatively, adequate environmental factors, which are produced by the white matter, might be required for proper differentiation of F3.Olig2 cells. F3.Olig2 cells also exhibited much higher capacity for migration towards the white matter than parental F3 cells. It is possible that demyelinating but intact axons may produce certain molecular cues to recruit oligodendrocyte lineage cells from the transplantation sites [39]. Most of the parental F3 NSCs without Olig2 overexpression, however, might not possess capacity to respond to such a signal. These observations may exemplify the importance of an adequate crosstalk between cell-autonomous traits of transplanted cells and host environmental factors for the successful integration of exogenous cellular grafts.

Transplantation of F3.Olig2 NSCs significantly increased the volume of myelinated white matter. Furthermore, animals with F3.Olig2 grafts showed significantly higher myelin ratio in the spared white matter. These data suggest a possibility that F3.Olig2 cells differentiated into functional oligodendrocytes and participated in remyelinating denuded axons in the residual ventrolateral white matter. It is also possible that F3.Olig2 NSCs in the spared white matter exerted trophic effects [40], which could prevent further demyelination and promote endogenous remyelination process [41]. Our finding that F3 NSC grafts, which rarely differentiated into oligodendrocytes, also increased myelin ratio to some extent supports the notion that the trophic mechanisms were indeed in operation. Therefore, the higher myelin ratio in animals with transplantation of F3.Olig2 NSCs could be accounted for by both direct remyelination and trophic effects by the grafted cells.

We found in the present study that the overall number of F3.Olig2 cells detected at 7 weeks after transplantation was larger than that of NSCs across the rostrocaudal extent of the spinal cord examined. A recent study has reported that Olig2 critically regulates replication competence in neural stem cells and malignant glioma [30]: for example, loss of Olig2 resulted in a dramatic reduction of neural stem cell proliferation. Our data also demonstrated that Olig2 overexpressing human NSCs possess higher proliferative capacity *in vitro *and *in vivo*. Therefore, the larger number of F3.Olig2 cells detected after the transplantation could be explained by the influence of Olig2 gene on proliferation potential of NSCs. Despite the proliferation-promoting effect of Olig2 gene, we did not observe a gross tumor formation. This is consistent with the fact that Olig2 is not sufficient for brain cancer formation [30]. It is conceivable that the increase in the number of grafted cells may have contributed to a better preservation of spinal cord tissue in animals with F3.Olig2 grafts. Many of F3.Olig2 or F3 cells that stayed in the gray matter or around the lesion cavity did not acquire any mature phenotype. These undifferentiated progenitor cells can exert neuroprotective effects by paracrine actions from secreted molecules or via an immunomodulatory mechanism [42,43]. Furthermore, undifferentiated NSCs can promote axonal sprouting/regeneration by producing neurotrophic factors or acting as a kind of cellular guidance [44,45]. F3 human NSCs have been shown to produce various neurotrophic factors [26,28]. The presence of a much larger number of grafted NSCs in F3.Olig2 group could promote tissue sparing or axonal growth of a much larger magnitude, which then resulted in the reduction of cavity volume. It is likely that the neuroprotective and/or neuroregenerative mechanism by grafted F3.Olig2 cells, together with enhanced myelination in the white matter, contributed to improvement in the quality of locomotion.

The animals grafted with F3.Olig2 cells improved quality of hindlimb locomotion as assessed by BBB score and footprint analysis. They exhibited better coordination between hind- and forelimbs. The smaller distance between the two hindlimbs (interlimbs distance) in animals with F3.Olig2 grafts suggests an improved balance during locomotion. However, there was no difference in the number of errors in grid walk test between the treatment groups. Walking on the grid runway requires precise motor control (hindlimbs placement) integrated with sensory information on the grid and thus depends on the integrity of supraspinal projections more heavily than spontaneous locomotor behavior [46,47]. Regaining supraspinal control may not be achieved merely by enhancement of myelination without regeneration of severed axons. In this regard, it may be necessary to combine strategies that are aimed at inducing axonal regeneration to achieve more meaningful functional improvement after SCI.

Several studies have highlighted the therapeutic benefits of supplying new oligodendrocytes by transplantation of stem/progenitor cells following SCI [11,12,15,48]. Transplantation of retinoic acid-treated or predifferentiated embryonic stem cells resulted in differentiation into oligodendrocyte lineage and improvement of behavioral recovery after SCI [12,48]. Although no tumor formation was reported, the possibility of teratocarcinoma formation in a longer term still raises a safety issue in using embryonic stem cells. Transplantation of multipotent or glial lineage-restricted neural stem/progenitor cells also successfully promoted myelination and enhanced functional recovery [11,15]. However, the efficiency of oligodendrocyte differentiation might be unsatisfactory or additional measures would be needed to improve the differentiation efficiency [49]. The present study is the first to utilize a molecular factor governing the fate determination of oligodendrocytes to enforce oligodendrocytic differentiation following SCI. The beneficial outcomes of the current approach would provide a promising alternative to supply myelinating oligodendrocytes for spinal cord repair.

## Conclusion

The present study showed that transplantation of human NSCs genetically modified to express Olig2 transcription factor into the contused spinal cord enhances the extent of myelination in the spared white matter and improved locomotor recovery. Transplantation of neural stem/progenitor cells genetically modified to differentiate into oligodendrocytic lineage may be an effective strategy to improve functional outcomes following traumatic injuries to the spinal cord. Our study further suggests that manipulation of molecular factors governing cell fate decisions during development can influence the fate of grafted neural stem/progenitor cells and positively affect the reparative potential of the transplantation therapy.

## Methods

### Culture of human neural stem cells

Primary dissociated cell cultures of fetal human telencephalon tissues of 14 weeks gestation were prepared as described previously [23,50,51]. The cells were grown in T25 flasks in Dulbecco's modified Eagle medium (DMEM; HyClone, Logan, UT), supplemented with high glucose, 5% fetal bovine serum (FBS), 20 μg/ml gentamicin (Sigma, St Louis, MO), and 2.5 μg/ml amphotericin B (Sigma). The medium was changed twice a week. The permission to use the fetal tissues was granted by the Clinical Research Screening Committee involving Human Subjects of the University of British Columbia, and the fetal tissues were obtained from the Anatomical Pathology Department of Vancouver General Hospital.

PA317 amphotropic packaging cell line was infected with the recombinant replication-incompetent retroviral vector pLNX.v-myc, and the supernatants from the packaging cells were used to infect NSCs in human fetal telencephalon cultures. Stably transfected colonies were selected by neomycine resistance. Several stable clones of human NSCs were isolated, and one of them, HB1.F3 (F3 hereafter), was expanded for the present study. F3 human NSCs express ABCG2, nestin, and Musashi1, which are cell type specific markers for NSCs [28,52,53].

To generate Olig2 overexpressing human NSC line (F3.Olig2), Olig2 cDNA (a generous gift from Dr. Takebayashi, Okazaki, Japan) was ligated into multiple cloning sites of the retroviral vector pLPCX. PA317 amphotropic packaging cell line was infected with the recombinant retroviral vector, and the supernatants from the packaging cells were added to the F3 cells. Stably transfected colonies were selected by puromycine resistance.

### RT-PCR and immunocytochemistry

For RT-PCR analysis, NSCs were grown on poly-L-lysine coated Petri dishes in DMEM with 2% FBS for 3 days. Total RNA was extracted from cultured cells using Trizol (GIBCO-BRL). One μg of total RNA was reverse-transcribed into first-strand cDNA using oligo-dT primer (Promega, Madison, WI). For PCR amplification, specific primer pairs were incubated with 1 μl of cDNA in a 20 μl reaction mixture containing Taq polymerase. The sequences of the primers were as follows; AAATCGCATCCAGATTTTC/CACTGCCTCCTAGCTTGTC for Olig2, TCTACGACAGCAGCGACAAC/CTTGGAGCTTGAGTCCTGAG for Nkx2.2, CATGACCACAGTCCATGCCATCACT/TGAGGTCCACCACCCTGTTGCTGTA for GAPDH.

To examine phenotypic differentiation, human NSCs were grown on poly-L-lysine coated 9 mm Aclar fluorocarbon plastic coverslips in DMEM with 2% FBS for 5 days. Cells were washed three times with phosphate buffered saline (PBS) and then fixed with 4% paraformaldehyde for 10 minutes. After blocking with 5% normal goat serum, cells were incubated with primary antibodies for 2 hours at room temperature (RT). The following primary antibodies were used to examine the expression of neural cell phenotypic markers; anti-O4 (1:5; mouse monoclonal; Kim Lab), anti-galactocerebrosidase (GalC) (1:5; mouse monoclonal; Kim Lab), anti-myelin basic protein (MBP) (1:500; rabbit polyclonal; Chemicon, Temecula, CA), anti-glial fibrillary acidic protein (GFAP) (1:500; rabbit polyclonal; Chemicon), and anti-MAP2 (1:200; rabbit polyclonal; Chemicon). The coverslips were then incubated with appropriate secondary antibodies tagged with Alex-488 or Alexa-594 fluorophores (Molecular probes, Eugene, OR) for an hour. The nuclei were stained with 4', 6-diamidino-2-phenylindole (DAPI; Sigma) before mounting on the slides.

### Proliferation assay

Growth rate of NSCs was determined by measuring viable cell numbers at different time points after plating. The number of cells was measured by Cell Counting Kit-8 (Dojindo Laboratories, Kumamoto, Japan) according to the manufacturer's instructions. Briefly, cells were dispensed as 100 μl of cell suspension (1000 cells/well) in a 96-well plate. Ten μl of CCK-8 solutions were added to each well at different time points (0, 6, 12, 18, 24, 48, and 72 hours), and the plate was incubated at 37°C for 4 h. The absorbance value at 450 nm wavelength was measured with a dual-beam microtiter plate reader. The experiment was replicated four times with triplicate samples included in each experiment.

For 5-bromo-2'-deoxyuridine (BrdU) incorporation experiment, 100 μl of cell suspension (1000 cells/well) was plated on a 9 mm coverslip and incubated for 36 hours. BrdU (final concentration 2 μM, Sigma) was added to each well for 2 hours followed by immunocytochemistry as described above using anti-BrdU (1:500; rat monoclonal; Oxford, UK). Counting was performed in three randomly selected fields (×200) and the average value was obtained from three coverslips for each condition. The percent of BrdU positive cells out of all DAPI stained nuclei was obtained as BrdU incorporation index.

### Animals and surgery

Adult female Sprague Dawley rats (250 - 300 gram, Orient Bio Inc. Seongnam, Korea) were used in this study. All rats were maintained on a 12:12 hour dark and light cycle with food and water provided *ad libitum*. After being anesthetized with 4% chloral hydrate (10 ml/kg, injected intraperitoneally), rats were subjected to a dorsal laminectomy at the 9th thoracic vertebral level (T9-10) to expose the dorsal surface of the spinal cord. The NYU spinal cord impactor was used to inflict a standardized contusion; a 10 g impactor head was dropped from a height of 12.5 mm onto the exposed T9-10 spinal cord. Muscles and subcutaneous tissues were sutured in layer, and the skin was stapled. Sham operation involved only laminectomy without contusion on the spinal cord. The bladder was manually expressed twice daily until the animals resumed self voiding. Seven days after spinal injury, the spinal cord was reexposed for cellular transplantation. Animals were randomly divided into three groups: 1) vehicle (PBS) injected (Vehicle group), 2) transplantation of F3 cells (F3 group), and 3) transplantation of F3.Olig2 cells (F3.Olig2 group). Two injections were made at 2 mm rostral and caudal to the epicenter using a glass micropipette (tip diameter < 70 μm) configured with Hamilton syringe. The pipette pierced the dorsal spinal cord slightly off the dorsal median sulcus, avoiding blood vessels at the midline. We advanced the pipette with a depth of 1.2 mm from the dorsal surface and kept it for 3 minutes during the injection which was controlled by Nanoliter syringe pump (KD scientific; Holliston, MA, USA). Each injection consisted of 1 × 10^5 ^of the dissociated cells in 2 μl of PBS. Thus, a total of 2 × 10^5 ^cells were transplanted for each animal. To prevent leakage from the injection site, the pipette was maintained for three more minutes and then withdrawn slowly. Animals were assigned with new identification codes after transplantation to ensure blind evaluation of behavioral performance. All animals received daily intraperitoneal injection of cyclosporine (Sandimmun; Novartis, Bern, Switzerland) at a dosage of 10 mg/kg, beginning from one day prior to transplantation to three weeks after transplantation. After that, cyclosporine (50 μg/ml) was administered through drinking water until animals were sacrificed. Prophylactic antibiotics were intraperitoneally injected on the next day after each surgery.

### Assessment of locomotor recovery

A total of 35 animals (N = 11, 12, and 12 for Vehicle, F3, and F3.Olig2 groups, respectively), divided in three series, underwent behavioral tests to assess locomotor recovery. The BBB (Basso, Beattie, and Bresnahan) locomotor rating scale was used to assess the extent of locomotor recovery during open field locomotion. Two experimenters who were blind to experimental conditions scored BBB scale separately and the average of the two scores was obtained. The grid walk test was conducted at 4 and 7 weeks after initial injury. We performed grid walk test for the second and third series of animals, so the animal in the first series were excluded (total 5; Vehicle = 1, F3 = 3, F3.Olig2 = 2). For grid walk, rats were trained to cross a grid runway (30 cm × 140 cm with 50 × 50 mm holes) for a water reward. Two animals in Vehicle group and one animal in F3.Olig2 group could not be trained adequately enough to undergo grid walk test. Therefore, grid walk data were obtained from 27 rats (Vehicle = 8, F3 = 9, F3.Olig2 = 9). On the day of test, four runs were recorded using a three-CCD digital video camera (NV-GS250, Panasonic, Japan), and later analyzed frame by frame in slow motion. The average number of limb placement errors per run was obtained for each animal. For footprint analysis, rats were trained to walk on a runway with a narrow alley made of transparent Plexiglas (7 cm × 170 cm) on top for a water reward during the same training session for grid walk. The animals' hindpaws were inked and footprints were obtained on white paper covering the floor of the runway. The base of support, which is a distance between the two hindlimbs, was determined by measuring the distance between the central pads of both hindpaws. Right and left stride lengths were measured between two consecutive prints on each side. Footprint analysis was performed only in the third series of animals (total 14; Vehicle = 4, F3 = 5, F3.Olig2 = 5). Normal foot print data were obtained from 5 rats with sham operation.

### Histological processing and immunohistochemistry

For histological analysis, animals were anesthetized with an overdose of chloral hydrate and perfused with heparinized saline (0.9%), followed by 4% paraformaldehyde in 0.1 M phosphate buffer, pH 7.4. The spinal cord was dissected and post-fixed in 4% paraformaldehyde at RT for 2 hours, followed by cryoprotection in a graded series of sucrose solutions (10%-30%) in 0.1 M phosphate buffer at 4°C. Transverse sections (20 μm) of the spinal cord were cut using cryostat (Leica CM 1900; Wetzlar, Germany) in a 1:10 series and thaw-mounted onto silane-coated glass slides. To quantify the area of myelinated white matter, transverse spinal cord sections were stained with eriochrome cyanine that stains myelinated white matter [54]. The transverse sections were immersed for 8 minutes in the staining solution consisting of 240 ml of 0.2% eriochrome cyanine RC (Sigma) and 10 ml of 10% FeCl_3_·6H_2_O (Sigma) in 3% HCl. The sections were then washed with running tap water, followed by differentiation in 1% aqueous NH_4_OH.

For immunohistochemistry, transverse spinal cord sections were incubated overnight at 4°C with anti-human mitochondria (1:400; mouse monoclonal; Chemicon), anti-Ki-67 (1:500; rabbit polyclonal; Novocastra, Newcastle, UK), anti-myelin basic protein (MBP) (1:400; rabbit polyclonal; Chemicon), anti-APC-CC1 (1:200; mouse monoclonal; Calbiochem, La Jolla, CA), anti-GFAP (1:500; rabbit polyclonal; Chemicon), anti-nestin (1:500; rabbit polyclonal; Chemicon), and anti-MAP2 (1:200: rabbit polyclonal; Chemicon). The spinal cord sections were washed and then incubated with biotinylated or fluorophore-tagged secondary antibodies. For chromogenic detection of antigen-antibody reaction, preformed avidin-biotinylated peroxidase complexes were applied for 30 minutes, followed by incubation with peroxidase substrate (DAB) until desired intensity developed. For fluorescence staining, coverslips were mounted onto glass slides using Gelvatol and examined under Olympus confocal laser scanning microscope (Model FV 300, Tokyo, Japan).

To analyze the thickness of myelin sheath wrapping individual axons, a separate series of animals (16 rats; Vehicle = 3, F3 = 6, F3.Olig2 = 7) were perfused with modified Karnovsky's fixative solution (2% glutaraldehyde and 1% paraformaldehyde in 0.1 M cacodylate buffer). Dissected spinal cord was divided into smaller blocks with about 5 mm in length. The tissue block containing caudal regions to the epicenter was transversely sectioned into 200 μm slices with a vibratome (model Vibratome Series 1000; Technical Products Int'l. Inc., O'Fallon, MO). The slices containing the regions around 1 mm caudal to the epicenter were immersed in 1% OsO_4 _solution, and then embedded in Poly/Bed 812 embedding media (Polysciences Inc., Warrington, PA). Transverse semithin (1 μm) sections were cut from the rostral surface with glass knife and then stained with toluidine blue.

### Quantitative cell counting and image analysis

Human mitochondria positive cells (developed with DAB substrates) were counted using unbiased stereology. Two transverse sections 200 μm apart from each other at the epicenter, ± 1 mm, ± 2 mm, ± 3 mm, and ± 4 mm rostral and caudal from the epicenter were chosen. Cell counting was performed on an Olympus BX51 Microscope with a MAC 6000 Motorized Stage Encoder System that was coupled with a computer running Stereo Investigator 8 software (MBF Bioscience, Williston, VT). Human mitochondria positive cells within optical dissectors randomly placed in regions of interest were counted using standard stereological criteria for inclusion. Stereological estimates were done in a systematic way using the formula in the software. The average of the two sections at each level was obtained for each animal. The percentage of the cell number in the spared ventrolateral white matter was calculated by dividing the number of cells in the ventrolateral white matter by the total number in the entire section. To determine the percentage of grafted human NSCs that were colocalized with proliferation marker Ki67 or neural cell specific markers, two transverse sections with the highest graft survival were chosen for each animal. The number of human NSCs colocalized with these markers was counted in the entire section and divided by the total number of human mitochondria positive cells. The average from the two sections was calculated as the final percentage value for each animal.

The volume of myelinated white matter and cystic cavities were determined for the animals that completed behavioral tests. Every 10th eriochrome-stained transverse spinal cord sections were viewed on an optical microscope (Olympus BX41). The section containing injury epicenter was defined visually as the one with a smallest visible rim of spared myelin. Then, serial sections with an equal distance (400 μm) spanning ± 2 mm from epicenter were imaged at a 40× magnification and captured with CCD camera (Olympus DP11). Myelinated spared ventrolateral white matter anterior to the dorsal horn was drawn with the Pen Tablet input device (Bamboo MTE-450K; Wacom Co., Tokyo, Japan) on each image and the cross-sectional areas were measured using publicly available Image J software (NIH, Bethesada, MD). The total estimated volume was calculated using the Cavalieri's Principle. The individual subvolumes were obtained by multiplying the cross-sectional area by the distance between sections, and the subvolumes were summed to generate the total volume of spared white matter (∑_n _[cross sectional areas × intersection distance], n = number of sections analyzed). The volume of cystic cavities was calculated by the same equation.

Thickness of myelin sheath in individual axons was determined on toluidine blue stained transverse semithin sections. Two sections with adequate preparation were selected for each animal. In order to have corresponding regions imaged across all the animals, a single image field (120 μm × 90 μm) per section was located in the ventral white matter just anterior to the ventral gray horn by an independent experimenter who was blind to the experimental conditions. Images were taken at 1000× magnification using a Zeiss Axiophot upright microscopy equipped with Axiocam HR digital camera. Horizontal grid lines with a 10 μm interval were drawn using Photoshop software and the same grid lines were applied to all images. Only the axons that were intersected by the grid lines were included for quantification. The thickness of myelin sheath and the shortest axon diameter were measured using the Image J software. According to the previously published report by Karimi-Abdolrezaee et al. (2006) [15], the myelin ratio was obtained by dividing the total axonal diameter including thickness of myelin sheath by the diameter of axonal fiber excluding myelin sheath.

### Statistical methods

Statistical analysis was performed with SPSS version 12.0 (Chicago, IL, USA) or GraphPad Prism software version 4.0 (San Diego, CA, USA). The unpaired Student T test or one-way ANOVA followed by Tukey's *post hoc *test was used for statistical comparison of group means. Repeated measures two-way ANOVA was used to compare the number of cells or the percent cells in the white matter at different regions and BBB locomotor scores at multiple time points.

## List of abbreviations

SCI: (spinal cord injury), NSC: (neural stem cell); CNS: (central nervous system); PBS: (phosphate buffered saline)

## Authors' contributions

DHH carried out in vivo studies, participated in experimental design, helped in drafting the manuscript. BGK conceived the study, helped in acquisition and interpretation of data, wrote the manuscript, and gave final approval of the version to be published. EJK carried out in vitro studies and participated in acquisition of in vitro data. SIL helped in conduction of experiments and participated in acquisition and interpretation of data. ISJ participated in study design and participated in critical reviewing of the manuscript. HSK participated in study design and interpretation of data. SS helped in acquisition and interpretation of data on myelination. SUK conceived the study, helped in writing and reviewing the manuscript, and gave final approval of the version to be published. All authors read and approved the final version of the manuscript.

## Supplementary Material

Additional file 1 **Immunocytochemical detection of differentiation into mature astrocytes or neurons**. F3 (A, C) or F3.Olig2 (B, D) cells were grown on coverslips in DMEM with 2% FBS for 5 days and then fixed with 4% paraformaldehyde. Then the cells were stained with anti-GFAP (A, B) or anti-MAP2 (C, D) antibodies. Some of F3 cells showed differentiation into astrocyte or neurons, but virtually no F3.Olig2 cells were immunoreactive against GFAP or MAP2. Click here for file
