# Supplementary figures and images for: Transplantation of human neural stem cells transduced with Olig2 transcription factor improves locomotor recovery and enhances myelination in the white matter of rat spinal cord following contusive injury
Source: BMC Neurosci. 2009 Sep 22;10:117. doi: 10.1186/1471-2202-10-117 (PMC2758886; doi:10.1186/1471-2202-10-117)

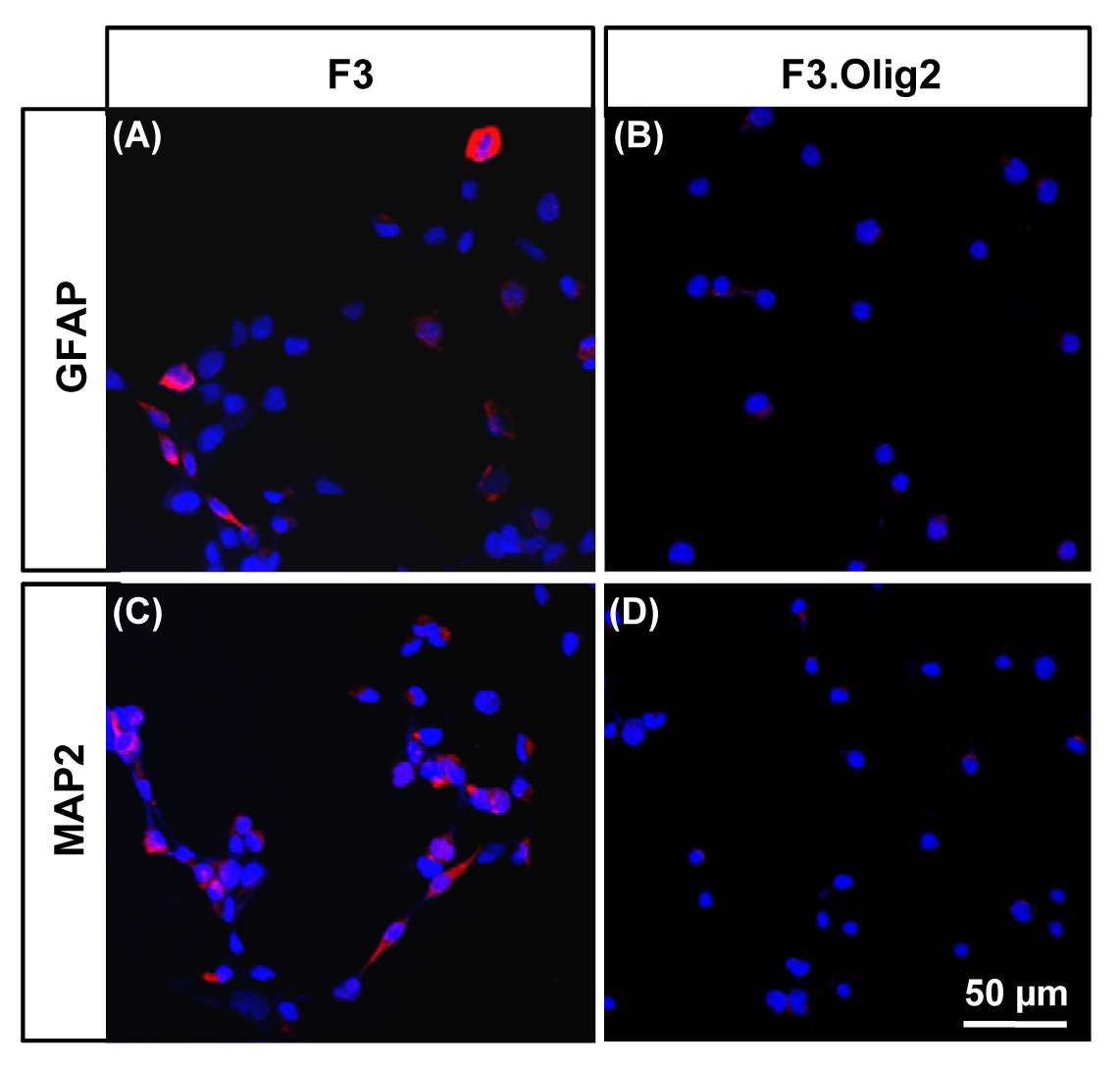

Supplement: Additional file 1 — Immunocytochemical detection of differentiation into mature astrocytes or neurons. F3 (A, C) or F3.Olig2 (B, D) cells were grown on coverslips in DMEM with 2% FBS for 5 days and then fixed with 4% paraformaldehyde. Then the cells were stained with anti-GFAP (A, B) or anti-MAP2 (C, D) antibodies. Some of F3 cells showed differentiation into astrocyte or neurons, but virtually no F3.Olig2 cells were immunoreactive against GFAP or MAP2. [file 1471-2202-10-117-S1.TIFF]
